# Can supplementation with vitamin C and E alter physiological adaptations to strength training?

**DOI:** 10.1186/2052-1847-6-28

**Published:** 2014-07-05

**Authors:** Gøran Paulsen, Kristoffer T Cumming, Håvard Hamarsland, Elisabet Børsheim, Sveinung Berntsen, Truls Raastad

**Affiliations:** 1 Department of Physical Performance, Norwegian School of Sport Sciences, Oslo, Norway; 2 Norwegian Olympic Sport Center, Oslo, Norway; 3 University of Arkansas for Medical Sciences, Arkansas Children’s Nutrition Center, Arkansas Children’s Hospital Research Institute, Little Rock, Arkansas, USA; 4 Department of Public Health, Sport and Nutrition, Faculty of Health and Sport Sciences, University of Agder, Kristiansand, Norway

**Keywords:** Protocol paper, Antioxidants, Muscle mass, Muscle strength, 1 repetition maximum

## Abstract

**Background:**

Antioxidant supplementation has recently been demonstrated to be a double-edged sword, because small to moderate doses of exogenous antioxidants are essential or beneficial, while high doses may have adverse effects. The adverse effects can be manifested in attenuated effects of exercise and training, as the antioxidants may shut down some redox-sensitive signaling in the exercised muscle fibers. However, conditions such as age may potentially modulate the need for antioxidant intake. Therefore, this paper describes experiments for testing the hypothesis that high dosages of vitamin C (1000 mg/day) and E (235 mg/day) have negative effects on adaptation to resistance exercise and training in young volunteers, but positive effects in older men.

**Methods/design:**

We recruited a total of 73 volunteers. The participants were randomly assigned to receiving either vitamin C and E supplementation or a placebo. The study design was double-blinded, and the participants followed an intensive training program for 10–12 weeks. Tests and measurements aimed at assessing changes in physical performance (maximal strength) and physiological characteristics (muscle mass), as well as biochemical and cellular systems and structures (e.g., cell signaling and morphology).

**Discussion:**

Dietary supplements, such as vitamin C and E, are used by many people, especially athletes. The users often believe that high dosages of supplements improve health (resistance to illness and disease) and physical performance. These assumptions are, however, generally not supported in the scientific literature. On the contrary, some studies have indicated that high dosages of antioxidant supplements have negative effects on exercise-induced adaptation processes. Since this issue concerns many people and few randomized controlled trials have been conducted in humans, further studies are highly warranted.

**Trial registration:**

ACTRN12614000065695

## Background

Exercise and training have indisputably demonstrated impressive potential in inducing physiological adaptations in both the cardiovascular and muscular systems [1-4]. Nonetheless, researchers still strive to elucidate the most efficient ways to exercise for certain adaptations, such as increased strength and endurance. A prerequisite for optimal effects of training is adequate nutrition, and indeed, the intake of certain types and amounts of macro- and micro-nutrients may modulate the effects of training [5]. Whey protein and creatine supplementation are two examples [6,7]. Antioxidants constitute a central group of micro-nutrients. Several studies have been conducted to explore their effects on health, physical performance, exercise and training [8-13]. The human body definitely requires a variety of antioxidants, but an unanswered question is whether it is beneficial or not to supplement the diet with isolated, highly concentrated antioxidant products, such as vitamin C and E pills [10,14].

Supplementation with different types of antioxidants has been variably shown to have positive effects, no effect, and even negative effects on training adaptation [10,15-17]. Because intensive exercise generates stress in the working muscles [16], it seems logical that antioxidant supplementation could beneficially reduce this stress. On the other hand, it has become clear that cellular stress, which includes increased production of free radicals and oxidative stress, in fact works as a signal to induce important adaptions in muscle cells, including mitochondrial biogenesis and myofiber hypertrophy [13,18]. Thus, if the cells are exposed to high levels of antioxidants this signaling may be blunted or blocked, which in turn may inhibit physiological adaptations. In line with this, Makanae et al. [19] recently reported that high dosages of vitamin C attenuated hypertrophy of overloaded plantaris muscles in rats. The diminished muscle growth appeared to be related to reduced overload-induced phosphorylation of p70S6K. Similar human studies are currently lacking, but Morales-Alamo et al. [20] have recently reported that an antioxidant mix (vitamin C, E and alpha-lipoic acid) blunted the activation of Ca^2+^/calmodulin-dependent protein kinase II (CaMKII) and AMP-activated protein kinase after maximal sprint exercise. Such signaling could be important for initiating training adaptation, and could explain the abolished training effects observed in anaerobic training combined with antioxidant supplementation (Q-10; [21]).

Hitherto, most investigations on antioxidant supplementation and training in humans have focused on endurance training (for reviews, see [10,15,22]). Only three human training studies have applied a high-force, resistance mode of exercise; i.e., traditional strength training [23,24] and eccentric exercise [25]. In a study by Theodorou et al. [25], no effects of vitamin C and E supplementation (1000 mg and 400 IU, respectively) were found on either recovery after eccentric exercise or adaptation to 4 weeks of eccentric training. Chuin et al. [24] supplemented elderly women (Vitamin C: 1000 mg/d, vitamin E: 600 IU/d) who concomitantly participated in strength training or control groups. The authors only reported results on bone mineral content (BMC), which allegedly was preserved better with C and E vitamin supplementation, but there was no interaction between training and supplementation. Bobeuf et al. [23] observed that vitamin C (1000 mg/d) and E (400 IU/d) supplementation affected neither body composition nor strength gain in elderly participants. Notably, Bobeuf et al. found no clear muscle gain in the placebo group, which means that the antioxidant effect on muscle growth is uncertain.

The effect of antioxidant supplementation and exercise in elderly individuals is indeed elusive. However, Ryan et al. [26] observed that vitamin C and E supplementation improved concentric work capacity in old rats, but not young rats. This suggests that antioxidant supplementation could exert different effects in young and aged muscles; possibly because of low levels of inflammation and augmented oxidative stress levels in aged muscles [27,28]. Consequently, aged muscles seem more likely to respond positively to antioxidant supplementation [29].

In the present study, we aimed to investigate the effects of antioxidant supplementation, in the form of vitamin C and E supplementation, recruiting both young (20–40 years) and elderly (60–80 years) volunteers. The effects of both a training period (10–12 weeks) and an acute exercise session with recovery were examined. Biochemical analyses of blood and muscle were combined with physiological measurements and performance tests.

Based on the notion that antioxidants blunt exercise-induced redox sensitive signaling in young, healthy muscles, but reduce unfavorable oxidative stress in aging muscles, we hypothesized that vitamin C (1000 mg/day) and E (235 mg/day) supplementation would:

(1) inhibit the adaptation to strength training in young, healthy men and women; and (2) augment the adaptation to strength training in healthy, elderly men (60–80 years). Moreover, we hypothesized that “hypertrophic” cell signaling and protein synthetic rate would be blunted in the hours after a single session of strength training in young persons, and, thus, reflect the long-term effects.

## Methods

This study comprises three experiments and involves the potential interference of antioxidant supplementation in exercise-induced responses and adaptations to strength training in young and elderly participants.

### Participants

**Figure 1:**
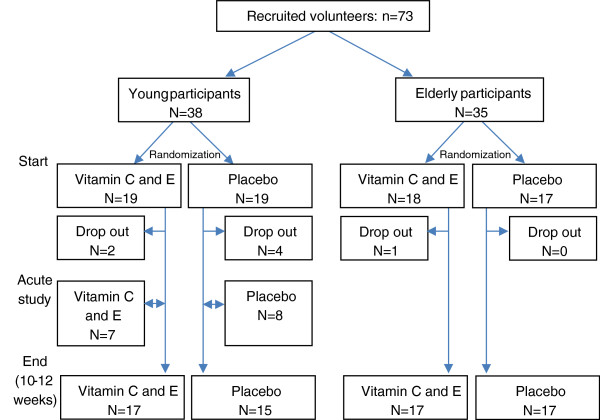
Flow chart of recruited and allocated participants.

A total of 73 participants were recruited to the study (Figure 1).

The young participants (20–45 years of age), both males and females, were healthy and did not use any form of supplements or medications, except contraceptives for women (menstrual cycle was not controlled for). They were all accustomed to strength training (recreationally trained), exercising 1–4 times per week. We excluded volunteers who did more than four strength training sessions per week to avoid including highly trained individuals, who are expected to show little or no progress over 10 weeks of training [4].

The older participants were males aged between 60–80 years. They were healthy, although some medications to treat mildly elevated cholesterol and blood pressure, migraine, and the use of mild antidepressants were accepted. The older participants were not engaged in strength training when they entered the study.

Interested volunteers who already used some kind of supplements, such as vitamins, were allowed to join the study if they immediately stopped using the supplements and went through a wash-out period of at least two weeks.

The young participants filled out a standard health form (developed at the Norwegian School of Sport Sciences), concerning health status and risk factors (smoking etc.). If a participant had a potential health issue, he or she was examined and cleared for training by a medical doctor before entering the study.

All elderly participants underwent a medical examination before entering the study, including blood pressure assessment, rest and work echocardiogram, as well as vascular ultrasound scans of the carotid arteries and aorta.

All participants signed a written informed consent. The study was approved by the South East Regional Committee for Medical and Health Research Ethics in Norway, and was performed in accordance with the Helsinki Declaration.

## Designs

**Figure 2:**
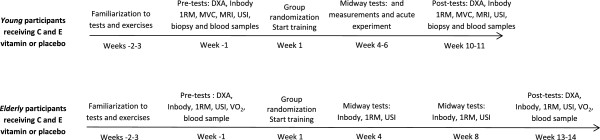
**Timeline for the experiment with young (upper line) and elderly participants (lower line).** DXA: Dual-energy X-ray absorptiometry, MRI: Magnetic resonance imaging, MVC: Maximal voluntary contraction (isometric), 1RM: 1 repetition maximum, USI: ultrasound imaging, VO2: oxygen uptake (work economy).

The study defined two training/exercise and antioxidant supplementation interventions:

1. Strength training and vitamin C and E supplementation with young participants

2. Strength training and vitamin C and E supplementation with elderly participantsTests and assessments were conducted before, during, and after the training periods (10–12 weeks). One to three familiarization sessions were conducted before the strength tests (see description below; Figure 2).

Baseline tests were conducted before group allocation so that the volunteers could be randomly assigned to receive a supplement or placebo, stratified for sex (for the young participants) and muscle strength (1 repetition maximum, 1RM). Research Randomizer (http://www.randomizer.org/) was applied to perform the randomization procedure. The experiments with young and elderly volunteers were performed separately at two different institutions.

Both experiments (young and elderly volunteers) were carried out in a double blinded fashion. The scientists responsible for the administration of the supplements were not involved in the tests and assessments.

The main outcome of the experiments with both the young and the elderly participants was a change in muscle mass, measured by dual-energy X-ray absorptiometry (DXA; see power calculations under “Statistics”). For assessing local muscle growth, magnetic resonance imaging (MRI) and/or ultrasound were applied. Bio-impedance measurement of body composition was used auxiliary to DXA and to provide midway measurements.

Secondary outcomes were measurements of muscle strength, measured by 1RM and isometric tests (maximal voluntary contraction; MVC), as well as analyses of tissue samples (young participants only). The tissue samples were used for analyses of endogenous antioxidant systems (e.g., glutathione peroxidase).

Additionally, blood samples were collected from all participants before, midway and after the intervention periods for analyses of endogenous and exogenous (vitamin C and E) antioxidant levels. Work economy during level/uphill walking was measured in the elderly participants only.

Assessments of nutrient intake and level of physical activity were undertaken in order to control for potential confounding factors.

### Acute experiment

**Figure 3:**
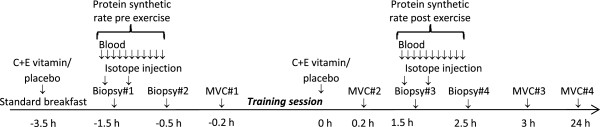
Timeline for the acute experiment with young participants.

After 4–6 weeks of training, an “acute” experiment, in the form of a single exercise session, was conducted with a randomly selected subgroup of the young participants (Figure 1).During the experiment MVC tests were conducted before and in the hours after the exercise session, for assessment of recovery of muscle function. Muscle and blood samples were obtained before and after the exercise session. Supplements were ingested ~3 hours before and immediately after exercise. Figure 3 displays the layout of the experiment.

The rationale for the acute experiment was to elucidate the acute cellular processes (signaling for hypertrophy), and potential correlations with long-term adaptations (increase in muscle mass).

The main outcome in the acute experiment was changes in the fractional synthetic rate of mixed muscle protein (protein synthesis).

Secondary outcomes were recovery of muscle function and assessments of the phosphorylation state of protein kinases (e.g., p70S6K) involved in “hypertrophic” intracellular signaling.

### Supplementation

#### Vitamin C and E supplementation

The vitamin C and E and placebo pills were produced under Good Manufacturing Practice (GMP) requirements at Petefa AB (Västra Frölunda, Sweden). Each vitamin pill contained 250 mg of ascorbic acid and 58.5 mg DL-alpha-tocopherol acetate (all-rac-alpha-tocopheryl acetate); in addition to cellulose, di/tri-calsium phosphate and magnesium stearate. Similar-looking placebo pills contained the same ingredients, except for the vitamins, and were produced by the same manufacturer. The pills were analyzed by a commercial company, Vitas (Oslo, Norway) ~2 years after production, with no sign of degradation of the vitamins (per pill: vitamin C: 255 ± 7 mg, vitamin E: 62 ± 2 mg). The experiments were conducted within this time period. No traces of the vitamins were found in the placebo pills.

The participants ingested two pills (totaling 500 mg of vitamin C and 117.5 mg vitamin E) 1–3 hours before every training session and two pills within the hour after training. On non-training days the participants ingested two pills in the morning and two pills in the evening. The intake of pills was confirmed in a training diary (see below). Thus, the daily dosage was 1000 mg of vitamin C and 235 mg vitamin E.

We divided the intake in two equal dosages to increase the bioavailability of the vitamins [30,31]. Moreover, due to the pharmacokinetics of the water-soluble vitamin C [32], the timing of the intakes was chosen to ensure that the blood levels of the antioxidants were high during exercise and in the first hours of recovery, when oxidative stress is suggested to be at its highest [16].

The participants were given pills for two weeks at a time, and exchanged the empty pill bottle for a new bottle every other week. The investigator who administrated the supplements did not take part in any of the tests or measurements, meaning that all tests were done blinded to group affiliation.

### Nutrition

#### Nutrition restrictions

Participants were asked not to take any form of nutritional supplement apart from those given in the study. They were asked to drink no more than two glasses of juice and four cups of coffee or tea per day. Juices especially rich in antioxidants, such as grape juice, were to be avoided.

### Standardized nutrient intake in the acute experiment

In preparation for the acute experiment, the participants ingested the supplements, C and E vitamin or placebo, followed by a standardized breakfast: 3 g oatmeal per kg body weight boiled in water with 10 g sugar, 2 hours before meeting in the laboratory.

#### Nutrition registration

The young participants completed a four-day weighed food registration dietary assessment [33] at the start and at the end of the training period. The participants used a digital food weighing scale (Vera 67002; Soehnle-Waagen GmbH & Co, Murrhardt, Germany; precision 1 g). The dietary registrations were analyzed using a nutrient analysis program (Mat på data 4.1; LKH, Oslo, Norway). We aimed to keep the participants in energy balance (or in slight positive balance), and to keep the intake of micro- and macro-nutrients, excluding the supplements, within the recommended ranges for Norway (http://www.helsedirektoratet.no).

Elderly participants registered their food intake using a four-day pre-coded food diary [34]. Diet recordings were performed during the same days as the activity recordings (see below). Daily intake of energy was computed using the food database and software system (KBS, version 4.9.) developed at the Department of Nutrition, University of Oslo, Norway.

A skilled nutritionist educated the participants in the given nutrient restrictions and in the diet registration prior to registration.

### Training diary

The participants filled out a training diary. On training days they recorded details about the training (exercises, kg lifted, etc.), as well as their subjective perception of effort during the sessions (Borgs scale [35]), and confirmed intake of pills. On non-training days they confirmed intake of pills and recorded muscle soreness, and if present, any symptoms of injuries/illness/sickness. Soreness and symptoms of injuries/illness/sickness were rated on a scale from 0–10, where 0 is no soreness/symptoms and 10 is extreme soreness and severe symptoms.

The training diary for the young participants was electronically available to the researchers during the training period, while the training diary for elderly participants was registered manually on paper and controlled during each exercise session.

### Training regimes

#### Young participants

The young participants followed a “classical” strength training program with heavy loads (6-11RM) and with short rest periods (1–1.5 min) for four sessions per week (Table 1). Exercises for all major muscle groups were included in a 4-split exercise routine (two upper body and two lower body sessions per week; Table 1). Variation in exercises for the different muscle groups was intended to enhance the exercise stimuli and reduce the risk of injuries. The main goal was to stimulate both increase in maximal strength and muscle growth.

**Table 1:** The training program for young participants

**Week**	**Load (RM)**	**Sets**	**Inter-set rest (min)**	**Sessions per week**
1-6	9-11	3	1	4
7-10	6-8	3-4	1.5	4
**Upper-body**	**Lower-body**	**Upper-body**	**Lower-body**	
**Exercises #1**	**Exercises #1**	**Exercise #2**	**Exercises #2**	
Bench press	Squat	Incline chest press	Deadlift	
Dumbbell flyes	Lunge	Pullover	Lunge	
Standing shoulder press	Knee-extension	Lateral rise	Leg press	
Triceps push-down	Straight leg deadlift	Pull-down narrow grip	Knee-flexion	
Seated row	Standing calf raise	Standing bent over row	Standing calf raise	
Pull-down wipe grip	Self-selected abdominal exercise	Biceps curl (scott curl)	Self-selected abdominal exercise	
Self-selected abdominal exercise		Self-selected abdominal exercise		

The young volunteers were supervised during the first sessions, and thereafter they had the opportunity to continue supervised training. However, most participants trained unsupervised after the initial supervision. This seemed acceptable, as most of the participants were familiar with strength training and the specific exercises before entering the study. Furthermore, as the recruitment period lasted for about 4 weeks, the volunteers stopped their current training routine and started to follow a simple whole body workout program with 3 sessions per week (8–12 repetitions with approximately 15RM loads), including the main exercises in the intervention program, until pre-testing and group randomization. Thus, this run-in period allowed the participants to become accustomed to the exercises used in the intervention period.

### Acute experiment

The exercise sessions consisted of four 10RM sets of leg press and knee-extension, with 1 min rest between sets and 3 min rest between exercises. The participants were verbally encouraged to execute each set to failure. If necessary, manual assistance was given so that the participants were able to complete the final repetition in each set.

### Elderly participants

The elderly participants followed a daily undulating (non-linear periodized) training program, with three sessions per week (Table 2). The total training volume increased during the first 10 weeks, but was reduced during the two last weeks (tapering). The elderly volunteers were unfamiliar with strength training, and all sessions were supervised by an investigator. As for the young, exercises for all major muscle groups were selected (Table 2), and the main aim was to increase maximal strength and muscle mass.

**Table 2:** The training program for elderly participants

	**Session 1**	**Session 2**	**Session 3**
Reps.	8-10	13-15	3-5
Rest	1 min	45 sec	2 min
Sets:			
Week 1	1 (warm-up) + 1	1 + 1	
Week 2	1 (warm-up) + 2		1 + 1
Week 3	1 (warm-up) + 2	1 + 2	
Week 4	1 (warm-up) + 2		1 + 2
Week 5	1 (warm-up) + 3	1 + 2	
Week 6	1 (warm-up) + 3		1 + 3
Week 7-8	1 (warm-up) + 3	1 + 3	1 + 3
Week 9	1 (warm-up) + 4	1 + 3	
Week 10	1 (warm-up) + 4		1 + 4
Week 11	1 (warm-up) + 3	1 + 3	
Week 12	1 (warm-up) + 2		1 + 2
	Exercises:		
	Bulgarian squat	“Sumo” deadlift w/kettlebells	Knee-extension
	Squat	Lunges	Leg press
	Bench-press	Step up	Chest-press
	Pull-down narrow grip	Flyes	Pull-down wide grip
	Upright row	Seated row machine	Arnold-press
	Calf raise	Lateral raises	Bench press narrow grip
	French press	Triceps pushdown	Scott curl
	Standing biceps curl w/dumbbells	Scott curl	Side-plank (abdominals)
	Quadruped exercise	Plank (abdominals)	

### Tests and measurements

#### Maximal strength testing of young participants

Maximal strength was assessed by 1 repetition maximum (1RM) tests and a maximal isometric voluntary contraction (MVC).

1RM was tested in knee-extension, knee-flexion, biceps curl and elbow extension. Each leg and arm was tested separately (unilateral tests). After a general warm-up (5 min walking or bicycling), a specific warm-up/preparation procedure was performed for each exercise, using loads corresponding to 50%, 70%, 80% and 90% of expected 1RM – conducting 10, 6, 3, and 1 repetitions, respectively. The loads were individually adjusted so the participants did not fatigue their muscles. Optimally, 2–5 attempts were used to establish 1RM. The loads could be adjusted in steps as low as 3%. The left and right leg/arm was tested interchangeably, so that each muscle rested for approximately 2 minutes between attempts. The range of motion was strictly controlled. The coefficient of variation (CV) of these assessments was <5%.

MVC was tested for the knee-extensors. The participants were fixed in a chair with belts over chest and hips, and an angle of 90° in the knee and hip joints. During the unilateral test the participants pressed maximally with their shank against the lever-arm of the apparatus, which was attached to a force-transducer (HBM U2AC2, Darmstadt, Germany). The participants got three 5-seconds attempts to reach MVC; 1 minute rest was given between attempts.

#### Maximal strength tests in elderly participants

1RM was tested in a similar manner as for the young participants (see above). 1RM was assessed in scott-curl (elbow flexors), leg press and knee-extension.

### Assessments of changes in muscle mass

#### Dual-energy X-ray absorptiometry, DXA

Body composition was measured by DXA (young participants: Hologic Discovery, Waltham, MA, USA; elderly participants: GE-Lunar Prodigy, Madison, WI, USA). Participants were scanned from head to toe in a supine position, providing values for bone mineral content, lean mass, and fat mass. The CV of these assessments was <2%.

### Bio-impedance

In addition to the body composition measurements by DXA, a bio-impedance apparatus was used (Inbody 720, Biospace Co., Ltd., Seoul, Korea). The apparatus has been found valid (compared with DXA) for estimating fat mass and lean mass in men and women [36]. Bio-impedance was used on both young and elderly participants.

### Magnetic resonance imaging (MRI)

MRI was only conducted on young participants. Transverse section images were captured of the dominant arm and both thighs (GE Signa 1.5 Tesla Echospeed, GE Medical Systems, Madison, Wisconsin, USA), before and after the intervention period. Thigh muscles: Joint gaps were used for reference points and nine images (5 mm) were captured with a 35.5 mm inter-image distance. Upper arm muscles: The os humerus of each participant was sectioned in nine evenly distributed images (5 mm). The images (DICOM) were analyzed using OsiriX 3.9.3 (Pixmeo, Bernex, Switzerland), giving the cross-sectional area of individual muscles. The CV of these assessments was <2%.

### Ultrasound imaging

In young participants, the muscle thickness and fascicle angle of m. vastus lateralis was assessed with a 50-mm linear probe (5–12 MHz) connected to a Philips HD11 XE ultrasound apparatus (Royal Philips Electronics, Amsterdam, Netherlands). In elderly participants, the muscle thickness of m. rectus femoris, m. vastus lateralis, and arm flexors (m. brachialis and m. biceps brachii) was assessed using a LogicScan 128 ultrasound apparatus (CEXT-1Z kit, Telemed Ltd., Vilnius, Lithuania) using a 60-mm probe. Fascicle angle was measured only in m. vastus lateralis. Image J software (National Institutes of Health, USA) was utilized to analyze the ultrasound images. The CV of these assessments was <5%.

### Work economy measurements of elderly participants

Work economy, determined as body mass adjusted oxygen consumption, was measured while walking on a treadmill (Woodway, Weil am Rhein, Germany), at 5 km/h at three different inclinations: 0%, 4% and 8%; 5 min at each inclination. Finger-stick capillary blood samples were collected and analyzed immediately for lactate concentration using a portable lactate analyzer (Lactate Pro, LT-1710, Arkay KDK, Kyoto, Japan) after finishing each inclination. Heart rate (RS800CX, Polar Electro KY, Kempele, Finland) was recorded continuously. Minute ventilation and respiratory exchange ratio (RER) were recorded continuously during each inclination using an Oxycon Pro (Jaeger BeNeLux, Breda, Netherlands). Equipment calibration was conducted before each test period.

### Physical activity level of elderly participants

Habitual physical activity was measured only in the elderly participants, and was measured with a SenseWear™ Pro3 Armband (BodyMedia Inc., Pittsburgh, PA, USA). The recording of physical activity started on a Saturday and included two weekdays and two weekend days. The monitor was worn on the right upper-arm at the midpoint between the acromion and olecranon processes as described previously [37]. Energy expenditure was computed at 1-minute intervals. Cut-off points defining moderate to very vigorous intensity were 3 metabolic equivalents (METs). Data from the monitor was downloaded and analyzed with software developed by the manufacturer (SenseWear Professional Research Software Version 6.1, BodyMedia Inc., Pittsburgh, PA, USA).

### Muscle tissue sampling and analyses

In young participants, a biopsy was sampled from the right leg before and after the 10 week training period (Figure 2). The participants who also took part in the acute experiment had four biopsies taken from the left leg (two biopsies before and two after the exercise session; Figure 3). Thus, each participant underwent no more than six biopsies.

Biopsies were taken from the mid-portion of m. vastus lateralis. The insertions for repeated biopsies were always located approximately 3 cm proximal to the previous insertion. The procedure was conducted under local anesthesia (Xylocain adrenalin, 10 mg/ml + 5 μg/ml, AstraZeneca, London, UK). Approximately 200 mg (2–3 × 50–150 mg) of muscle tissue was obtained with a modified Bergström technique. Biopsy samples intended for homogenization were quickly washed in physiological saline and fat, connective tissue, and blood were discarded, before being weighed and quickly frozen in isopentane cooled on dry ice. Muscle biopsies for immunohistochemistry were mounted in a Tissue-Tek compound (Cat#4583, Sakura Finetek, Torrance, CA, USA) and quickly frozen in isopentane cooled on liquid nitrogen. Tissue intended for mRNA analyses were placed in RNAlater (Cat#AM7020, Ambion, Life technologies, Carlsbad, CA, USA). All muscle samples were stored at −80°C for later analyses.

For immunohistochemistry, cross-sections were cut in a cryostat microtome (CM3050, Leica Biosystems GmbH, Wetzlar, Germany), and the samples were stained for dystrophin, SC71 (fiber type II) and CD56/NCAM for analyses of mean fiber area, fiber type distribution and satellite cells; DAPI was used to mark myonuclei (for more details regarding antibodies and procedures, see [38-40]).

Planned analyses by means of western blotting and ELISA, were: Heat shock protein (as previously described [41]), and endogenous antioxidants, such as MnSOD and glutathione peroxidation.

mRNA was analyzed using real time PCR, and generally, the mRNA analyses complemented the protein measurements (e.g., heat shock protein and endogenous antioxidants).

### Blood sampling and analyses

Blood was drawn from an antecubital vein into two 4.5 ml EDTA, one 4.5 ml Heparin, one 4.5 ml Stabilyte, and one 9 ml serum vacutainer tube. EDTA blood for hematological analyses was analyzed within 4 h after sampling. To extract plasma and serum the tubes were, after appropriate treatment, centrifuged at 1500 g for 10 min at 4°C. Plasma and serum were then pipetted into Eppendorf tubes and immediately stored at −80°C for later analyses.

Plasma destined for vitamin C analysis was mixed in equal volumes with metaphosphoric acid before storage at −80°C for later analyses. Plasma intended for glutathione analysis was treated in accordance with the procedure described by Sakhi et al. [42]. From the Stabilyte blood, erythrocytes were also collected for glutathione analysis. Other planned plasma/serum analyses included: cholesterol, triglycerides and CRP, plasma uric acid, creatine kinase and total antioxidant capacity (Modell X, MaxMat S.A., Montpellier, France), and various cytokines/chemokines (i.e., IL-6, IL-8, IL-10 and TNF-alpha; Milliplex MAP, Luminex xMAP technology by Millipore, Billerica, MA, USA).

### Protein synthesis

The procedure for measuring rate of protein synthesis is described in detail by Zhang et al. [43]. Briefly, a baseline blood sample (2 ml) was taken for background measurement of amino acid enrichment. Thereafter, a bolus injection of ^13^C_6_-phenylalanine was given at a dose of 50 μmol/kg, followed by a bolus of ^15^ N-phenylalanine (50 μmol/kg) 30 minutes later. All isotopes were from Cambridge Isotope Laboratories (Andover, MA, USA). Biopsies from m. vastus lateralis were collected at 10 and 50 min after the first bolus injection (see description of the biopsy procedure above). Muscle samples were quickly rinsed in cold saline, blotted and immediately frozen in liquid nitrogen and stored at −80°C for later analyses. A total of eleven blood samples (2 ml) were drawn during the procedure. Plasma was separated (as described above) and stored at −80°C for later analysis.

### Statistics

The primary outcome was a change in muscle mass (lean mass measured with DXA); with an expected gain of 1.5 kg in lean mass and a standard deviation of 0.7. Sixteen volunteers in each group gave 80% power to detect a difference of approximately 0.7 kg. (alpha: 0.05; two-tailed; StatMate, GraphPad Software, La Jolla, CA, USA).

The secondary outcome was a change in muscle strength (1RM); with an expected standard deviation (SD) of 15, we had 80% power to detect a group difference of 11% with 16 subjects in each group (alpha: 0.05; two-tailed; StatMate, Graphpad software).

The primary and secondary outcome variables were expected to be normally distributed and parametric tests were applied: Student t-tests for relative changes and 2-way ANOVA for comparing pre and post absolute values, as well as repeated measures. Outcome variables that turned out not to be normally distributed were analyzed with appropriate non-parametric tests.

## Discussion

In this study we aimed to test the hypothesis that high dosages of antioxidants, in the form of 1000 mg vitamin C and 235 mg of vitamin E (alpha-tocopherol) per day, will inhibit adaptation to strength training in young, healthy volunteers. In contrast, in elderly individuals (60–80 years), where the antioxidant status is expected to be lower, we tested the hypothesis that supplementation with vitamin C and vitamin E would augment the adaptations to strength training.

### The supplements

Exercise that challenges the energy systems of muscle cells induces metabolic stress, which includes oxidative stress by the generation of reactive oxygen and nitrogen species (RONS) [16,44]. Several endogenous antioxidants, such as glutathione, superoxide dismutase and catalase, will counteract most of the adverse effects of acute exercise. In addition, cells will take up exogenous antioxidants in the form of vitamins C and E, alpha-lipoic acid, carotenoids, ubiquinones and a range of phytochemicals [45], which will reinforce the cellular antioxidant system. In the present study we selected a vitamin C and E combination. Vitamin C and E are well established supplements and there appear to be no large risks concerning toxic effects, even if the daily dosages are several-fold higher than the recommended amounts [46,47]. Because the water soluble vitamin C and the lipid soluble vitamin E interact with the major endogenous antioxidant glutathione, a combination should be more effective than one single compound given alone. In short, vitamin C regenerates oxidized vitamin E, and oxidized vitamin C is in turn regenerated, although dependent on both the dose and the time elapsed between intake and measurement [32]. In contrast to vitamin E (alpha-tocopherol; [48]), the pharmacokinetics of vitamin C displays a quite rapid increase after oral intake and a gradual reduction during the next hours after intake [32].

Concerning the pharmacokinetics of vitamin C, the timing of intake in regard to exercise sessions should be considered. Some studies have administered supplements in the morning [23,25,49], and 1.5-2 hours before the exercise sessions [50]. Others have chosen two intakes per day [51,52]. In order to maximize the influence of the antioxidant supplementation, it thus seemed reasonable to time the intake so that the plasma concentration was highest just before and during the first hours of recovery after the exercise sessions.

We administered dosages of 1000 mg vitamin C and 235 mg of vitamin E (DL-alpha-tocopherol) per day. These amounts are in line with other recent studies, although our vitamin E dosages could be considered moderate [15].

### Participants

In the experiment with young volunteers we included both sexes. This was partly due to there being no obvious reasons to exclude one of the sexes, and partly because it is interesting to look for sex differences, as most studies on antioxidant supplementation and training adaptation have been conducted on males [25,49-54].

In the experiment on the elderly, we chose males only. The main reason for this was that very few studies have investigated interactions between exercise and antioxidant supplementation in elderly participants; however, Bobeuf et al. [23] have conducted studies on elderly males, and we wanted to follow up this study. The second reason was that elderly females appear to have less potential to respond to high intensity resistance exercise with three exercise sessions per week [55,56]. Thus, in order to increase the likelihood of inducing robust muscle growth, we selected elderly males.

We recruited recreationally trained young volunteers, but untrained elderly volunteers. Our motivation for choosing young, trained individuals was that we hypothesized that antioxidant supplementation would affect adaptations in the muscle. Thus, we assumed it was better to test this in trained individuals in whom muscular adaptations are expected to dominant over neural adaptations [57,58]. We chose untrained elderly volunteers, as we expected it would be very difficult to recruit the needed number of trained elderly participants. Furthermore, since we hypothesized that the elderly need more antioxidants to combat an increasing redox state in the aftermath of developing sarcopenia, we included untrained elderly individuals.

### High intensity exercise

Based on the theory of hormesis, the advantage of antioxidant supplementation should be largest during high intensity training that induces large oxidative stress; and conversely, low intensity training – that produces low oxidative stress – in combination with antioxidant supplementation should increase the likelihood of blunting or blocking training effects [18]. In our study we wanted to apply training programs that were similar to what dedicated strength-trained individuals use in practice. We therefore designed the training protocols to induce high metabolic and oxidative stress. Accordingly, the strength training program consisted of an energy-demanding, full range of motion exercises and repetition maximum (RM) intensity, as well as short inter-set/-exercise rest periods (see Table 1).

We designed a periodized strength training program, which has been suggested to be superior to non-periodized programs [59,60]. However, there is no consensus concerning what type of periodization is better [59]. For practical reasons, we designed a linear program for the young and an undulating (non-linear) program for the elderly.

### Limitations

Supplementation with vitamin C and E is a conservative choice, but the dosages chosen, 1000 mg vitamin C and 235 mg vitamin E (DL-alpha-tocopherol acetate), are in line with previously published studies [24,25,49-51,53,54]. Ideally, a dosage titration would be preferable (i.e., 400 mg, 800 mg and 1600 mg of vitamin C). Moreover, it was of interest to test vitamins C and E both separately and together; and in a stronger study design we would also include groups that received vitamins, but did not follow an exercise intervention. Unfortunately, we did not have the resources to design a study that took all these aspects into account.

We supplemented our participants with DL-alpha-tocopherol acetate, the synthetic form of vitamin E. It has been shown that the biological response to natural (D-alpha-tocopherol/RRR- alpha-tocopherol) may be different [61,62]. Thus, we must be careful when discussing our results alongside studies that have administered the natural form of vitamin E.

Training supervision was optional for the young participants in this study; while the elderly participants were supervised in all sessions. All participants were, however, instructed to keep a training diary in which they logged loads and perceived exertion, and they could request assistance whenever needed. Moreover, the young participants were familiar with strength training before entering the study. Nevertheless, full training supervision is preferable [63], to ensure that all participants follow the training as prescribed and exercise with appropriate lifting techniques and effort.

The training programs for young and elderly participants differed. This means that we cannot easily compare results from young and elderly participants. Such comparisons were not part of our hypotheses, but equal training programs could potentially give more information about the effects of the supplements. However, several practical reasons made us use different training regimes for the two groups of volunteers. Thus, we tried to optimize the training for the two groups independently of each other.

We monitored the influence of antioxidant supplementation over a rather short period of time, and long-term effects cannot be extracted. However, the most significant physiological changes occur typically in the first weeks after embarking on a new training program [58,64,57].

In the acute experiment we tested the response to a single session of strength training. The intention was to investigate the immediate cell signaling (e.g., redox sensitive mitogen activated kinases, MAPKs [65]) and the protein synthetic rate, in order to get a mechanistic insight into the hypothesized effects of the supplements. As the experiment was conducted midway into the intervention period, on the young participants, we realize that we cannot easily differentiate between the potential effects of the supplementation before/after the session and the potential effects of the preceding ~ 4 weeks with supplementation.

### Relevance for investigating the effects of vitamin C and E

Vitamin C and E supplements are widely used in the general population and even more so amongst athletes [66-71]. The most common motivation for using antioxidant vitamins such as vitamin C seems to be strengthening the immune system and thereby preventing and treating diseases, especially upper-respiratory infections such as the common cold [72]. Intriguingly, vitamin C supplementation seems at best to have limited clinically relevant effects on “the common man” and on athletes who are otherwise healthy and have a normal diet [72,73]. Some groups, such as the elderly population, are more susceptible to dietary deficiencies of some vitamins and therefore supplementation may be defensible [73,74].

Amongst athletes and people engaged in recreational sport activities, dietary supplements are also used to improve performance and recovery after exercise [9,71]. Equivocal observations have been reported, but vitamin C and E do not seem to have appreciable/consistent ergogenic effects or effects on recovery from exercise [9,14,75,76].

Regardless of the motivation for use, vitamin C and E supplementation is widespread. This fact, combined with recent studies that indicate adverse effects on adaptation to exercise and training [51,53], clearly illuminate and justify further research into the biological effects of vitamin C and E supplementation in humans.

## Conclusion

Antioxidant supplementations, such as vitamin C and E, are commonly used by large and divergent groups worldwide (e.g. athletes, elderly, patients etc.). Recently the potential interaction between antioxidant supplementation and training has received increased scientific interest and become a debated topic. Indeed, both animal and human studies have indicated negative effects when vitamin C and/or E are administered in high dosages. The literature is, however, ambiguous and many important questions remain unanswered. Hitherto, human investigations have focused on endurance exercise and training, so the experiments described here should provide novel data and insight into the interactions between antioxidant supplementation and adaptations to strength training.

## Competing interests

The authors declare that they have no competing interests.

## Authors’ contributions

GP contributed to all parts of the study. He currently works with data analysis and writing. KTC took part in the planning and design of the study with young participants. He contributed to the recruitment of volunteers and management of the experiments. KCT currently works with biochemical analyses of blood and muscle, as well as statistical data analysis and writing. KCT’s focus is on endogenous antioxidants and heat shock proteins. HH took part in the planning and design of the study with young participants. He contributed to the recruitment of volunteers and management of the experiments. HH currently works with biochemical analyses of muscle, as well as data analysis and writing. HH’s focus is on protein signaling after acute resistance exercise sessions. EB took part in the planning and design of the study with young participants. She had a special responsibility for the acute experiment concerning measurements of protein synthesis (stable isotope procedure). EB is currently involved in data analysis and writing. SB took part in the planning and design of the study with elderly participants. SB administered the study with the elderly participants, and is currently involved in data analysis and writing. TR contributed to all parts of the studies, and he was responsible for the biopsy procedure. He currently works with data analysis and writing. All authors read and approved the final manuscript.

## Pre-publication history

The pre-publication history for this paper can be accessed here:

http://www.biomedcentral.com/2052-1847/6/28/prepub
